# A robust cell culture system supporting the complete life cycle of hepatitis B virus

**DOI:** 10.1038/s41598-017-16882-5

**Published:** 2017-11-30

**Authors:** Eleftherios Michailidis, Jonathan Pabon, Kuanhui Xiang, Paul Park, Vyas Ramanan, Hans-Heinrich Hoffmann, William M. Schneider, Sangeeta N. Bhatia, Ype P. de Jong, Amir Shlomai, Charles M. Rice

**Affiliations:** 1 Laboratory of Virology and Infectious Disease, The Rockefeller University, New York, NY USA; 2 Department of Microbiology and Center of Infectious Disease, School of Basic Medical Sciences, Peking University Health Science Center, Beijing, China; 3 Department of Health Sciences and Technology, Massachusetts Institute of Technology, Cambridge, MA USA; 4 Institute for Medical Engineering and Science, David H. Koch Institute for Integrative Cancer Research, Department of Electrical Engineering and Computer Science, Massachusetts Institute of Technology, Cambridge, MA USA; 5 Howard Hughes Medical Institute, Cambridge, MA USA; 6 Division of Gastroenterology and Hepatology, Weill Cornell Medical College, New York, NY USA; 7 Department of Medicine D and the Liver Institute, Rabin Medical Center, Belinson Hospital, Petach-Tikva and Sackler Faculty of Medicine, Tel Aviv University, Tel Aviv, Israel

## Abstract

The discovery of sodium taurocholate cotransporting polypeptide (NTCP) as the hepatitis B virus (HBV) receptor enabled researchers to create hepatoma cell lines susceptible to HBV infection. Infection in current systems, however, is inefficient and virus fails to spread. Infection efficiency is enhanced by treating cells with polyethylene glycol 8000 (PEG) during infection. However, this alone does not promote virus spread. Here we show that maintaining PEG in culture medium increases the rate of infection by at least one order of magnitude, and, most importantly, promotes virus spread. To demonstrate the utility of this system, we show that two interferon-stimulated genes (ISGs), ISG20 and tetherin, restrict HBV spread in NTCP-expressing hepatoma cells. Thus, this protocol can be easily applied to existing cell culture systems to study the complete HBV life cycle, including virus spread.

## Introduction

More than 240 million people worldwide are chronically infected with hepatitis B virus (HBV) and are at high risk of developing liver cirrhosis and hepatocellular carcinoma^1,2^. Current HBV therapies, such as nucleoside analogs, suppress viral replication but they do not eliminate the virus. This is likely because the HBV genome persists in cells as a stable covalently closed circular DNA (cccDNA) for extended lengths of time^3,4^. As a result, prolonged and often life-long treatment is necessary to suppress viral replication and reduce the risk of cirrhosis and liver cancer^5^. In the clinic, this translates into high costs, possible adverse events and poor adherence. Developing novel antiviral strategies that lead to a functional cure is therefore a top priority of HBV research^6,7^.

Developing novel antiviral strategies requires efficient cell culture systems suitable for mechanistic studies and drug screens. A major milestone towards creating such systems was reached with the recent identification of sodium taurocholate cotransporting polypeptide (NTCP) as a receptor for HBV^8,9^. Now, by overexpressing NTCP in hepatoma cell lines like HepG2 and Huh7, one can study *de novo* HBV infection in simple and easy to use cell culture systems^8,9^. But unlike HBV infection *in vivo*, these cell culture-based systems are limited in their robustness and require high viral titers (ranging from 500 to 10,000 genome equivalents of HBV per cell) to achieve infection^8–11^. Infection can be increased by optimizing culture conditions, for example by including dimethyl sulfoxide (DMSO) in culture media^8,10^, and by spinoculation during infection^10^, but still, infection requires large quantities of virus and none of the available cell culture systems show evidence of virus spread. Thus, they fail to recapitulate the full HBV life cycle.

Polyethylene glycol 8000 (PEG) has been used for over 25 years to enhance HBV infection in cell culture systems including adult primary human hepatocytes, HepaRG hepatoma cells, and more recently HepG2-NTCP cells^8–10,12–14^. Schulze *et al*. showed that PEG promotes HBV binding to HepRG cells by increasing the interaction with heparan sulfate proteoglycans resulting in increased infection efficiency^14^. In all infection protocols PEG is mixed with the virus inoculum during the initial infection time (16–24 h) and then after washing out the inoculum fresh medium is added without PEG. In these systems, addition of PEG enhanced the initial infection efficiency but there was no detectable spread.

Even though there is no evidence of HBV spread in HepG2-NTCP cells, a recent study using iPS-derived hepatocyte-like cells (HLCs) infected with HBV showed measurable but minimal spread to neighboring cells^15^. This finding provides important proof of concept that HBV can spread in cell culture. However, the low efficiency of spread together with the high cost of generating and maintaining uniform HLC cultures make it difficult for large-scale screening applications. Therefore, more robust and easy-to-use infection systems that also support viral spread are still needed.

Here we report that continuous treatment with PEG following initial infection of HepG2-NTCP cells increases the efficiency of infection by supporting virus spread to uninfected cells. The efficiency of our system is such that we can detect at least one log increase of easily measurable HBV parameters within one week of the initial infection when PEG is continuously present. To our knowledge, this is the first report that shows productive infection with low starting inoculum concentrations (10 genome equivalents per cell - GEQ/cell) and efficient HBV spread in HepG2-NTCP cells. Our finding should be readily applicable to existing systems and will allow for drug screens as well as virus-host studies in the context of the full HBV life cycle.

## Results and Discussion

### Establishing an HBV infection system in HepG2-NTCP cells

**Figure 1:**
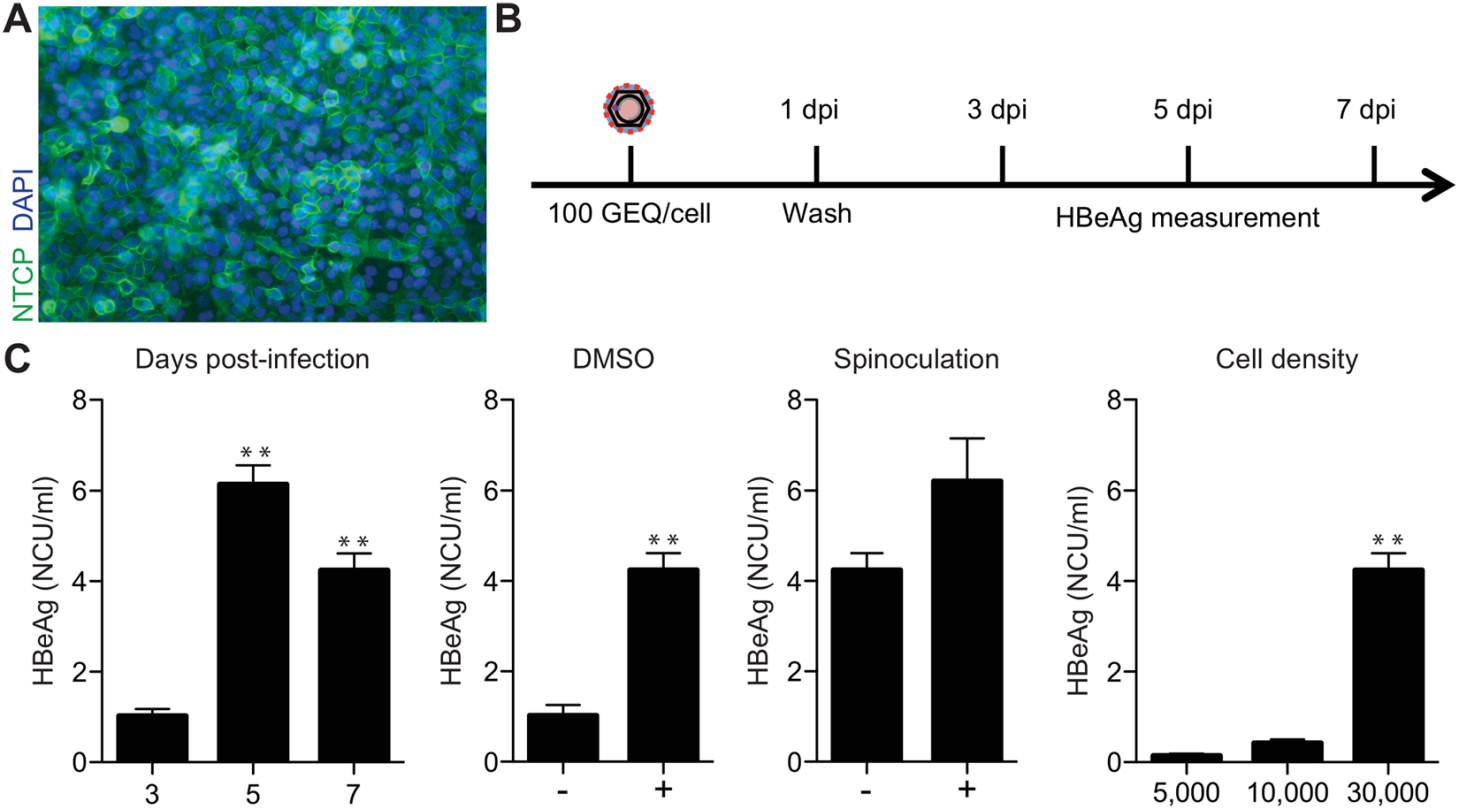
HBV infection and optimization in HepG2-NTCP cells. (**A**) Immunofluorescence microscopy to detect NTCP in HepG2-NTCP. (**B**) Schematic of the experimental design. (**C**) Optimization of HBV infection in HepG2-NTCP cells. Cells were infected with 100 GEQ/cell and the following conditions were optimized by measuring HBeAg in cell supernatants by ELISA X days post infection: duration of infection, DMSO treatment, spinoculation, and seeding cell density (96-well plate). In all conditions, 4% PEG was present in the inoculum, then washed out one day post-infection. At least three biological replicates were performed and data are shown as means ± s.d. (one-way ANOVA for days post-infection and cell density, two-tailed unpaired *t* test for DMSO and spinoculation, **P < 0.001).

To establish a cell culture system to study HBV infection, we first transduced HepG2 cells with lentiviruses expressing the HBV receptor, NTCP, and isolated single cell clones. Next, we used HBV produced by HepDE19 cells—a stable HBV-producing cell line that produces HBV from a genomic locus^16^—to screen for clones susceptible to HBV infection. The HepG2-NTCP cell clone most permissive to HBV was then used for the remainder of this study. As shown in Fig. 1A, NTCP was highly expressed on the surface of these cells.

We next optimized infection by varying conditions and measuring infection efficiency by HBeAg ELISA in a 96-well plate format. The experimental design is shown in Fig. 1B. The variables we optimized included cell density and duration of infection, presence or absence of 2% DMSO, and impact of spinoculation. Cells were infected with 100 genome equivalents per cell (GEQ/cell), and like all published protocols for infecting HepG2-NTCP cells with HBV, we included 4% polyethylene glycol 8000 (PEG) in the virus inoculum. After one day of infection, we washed cells 5 times with 200 μl of PBS per wash to remove virus and PEG, and then maintained cells in fresh medium for the duration of the experiment. Results are shown in Fig. 1C and Table 1. Based on HBeAg secretion 7 days post infection (dpi), we conclude that optimal infection requires confluent cell cultures on the day of infection, 2% DMSO treatment before and during infection, infection in the presence of 4% PEG, and spinoculation.

**Table 1:** HBV infection optimization using 100 GEQ/cell inoculum.

	HBeAg NCU/ml	Fold change
**Effect of DMSO and spinoculation during initial infection**		
Untreated	1.02	1
+2% DMSO	4.24	4
+2% DMSO + spinoculation	6.21	6
**Effect of duration of infection**		
3 dpi	1.03	1
5 dpi	6.14	6
7 dpi	4.24	4
**Effect of seeding density (cells/well)**		
5,000	0.13	1
10,000	0.42	3
30,000	4.24	33

Data represent average of five biological replicates.

**Figure 2:**
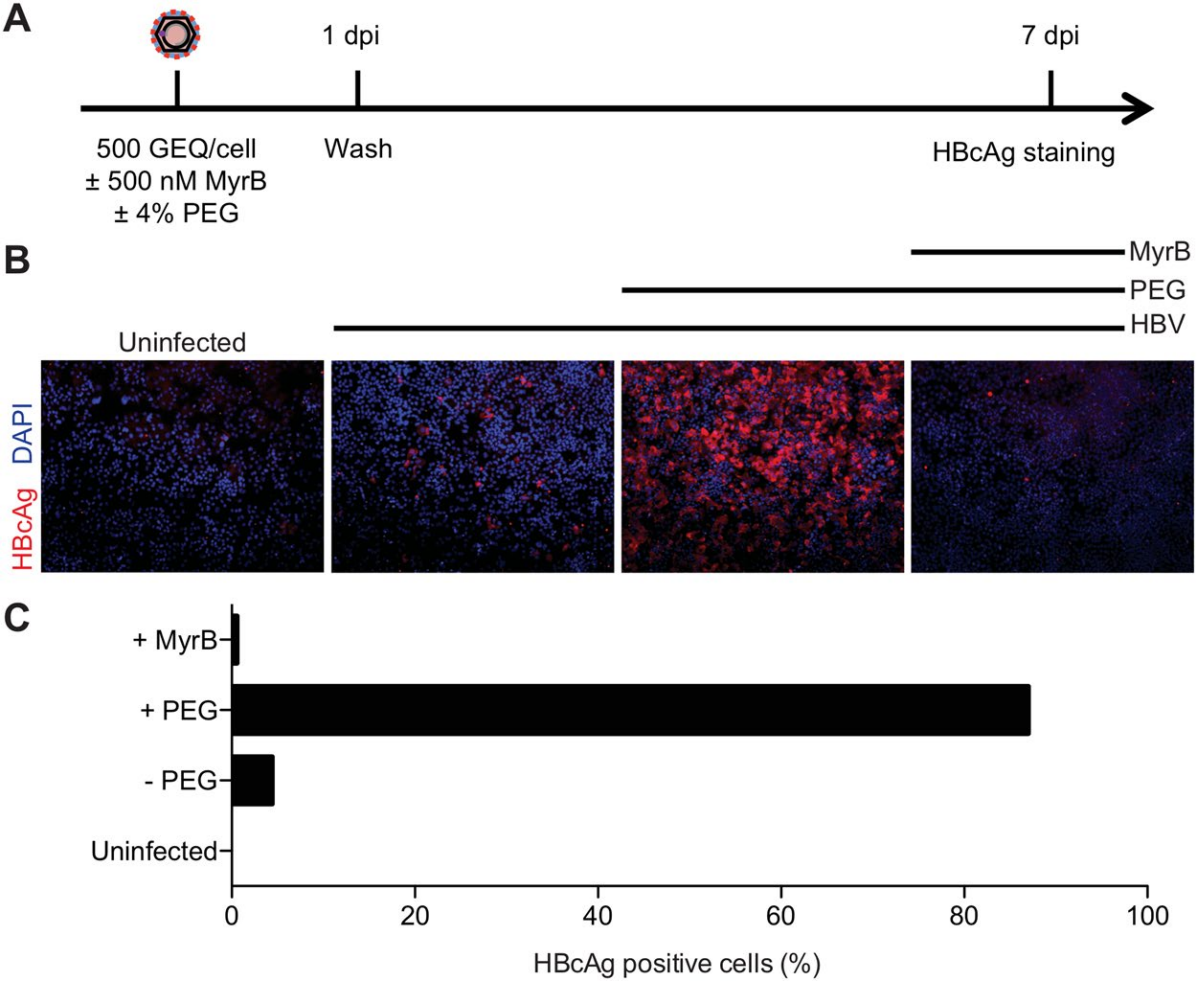
PEG enhances HBV infection in HepG2-NTCP cells. (**A**) Schematic of the experimental design. (**B**) Immunofluorescence microscopy to detect HBcAg in HepG2-NTCP cells 7 dpi. PEG substantially increased infection efficiency when added to virus inoculum for 24 h. 500 nM of HBV entry inhibitor, MyrB, prevented HBV infection. (**C**) Quantification of HBcAg positive cells under different conditions.

Next, we tested the impact of PEG on HBV infection. The experimental design is shown in Fig. 2A. For this we used our optimized protocol and infected cells for one day in the presence or absence of PEG, then, after 7 days, we assessed the frequency of infection by staining cells for HBcAg. To rule out the possibility that virus remaining from the inoculum contributed to the signal, we included a control where we pretreated cells with 500 nM Myrcludex B (MyrB), an inhibitor of virus entry^8^. As shown in Fig. 2B, MyrB potently inhibited infection and adding 4% PEG during infection significantly increased infection frequency. Quantification of HBcAg positive cells showed that more than 80% of the cells were infected in the presence of PEG as opposed to only 4% in the absence of PEG (Fig. 2C).

### HBV-infected HepG2-NTCP cells produce infectious viral particles

**Figure 3:**
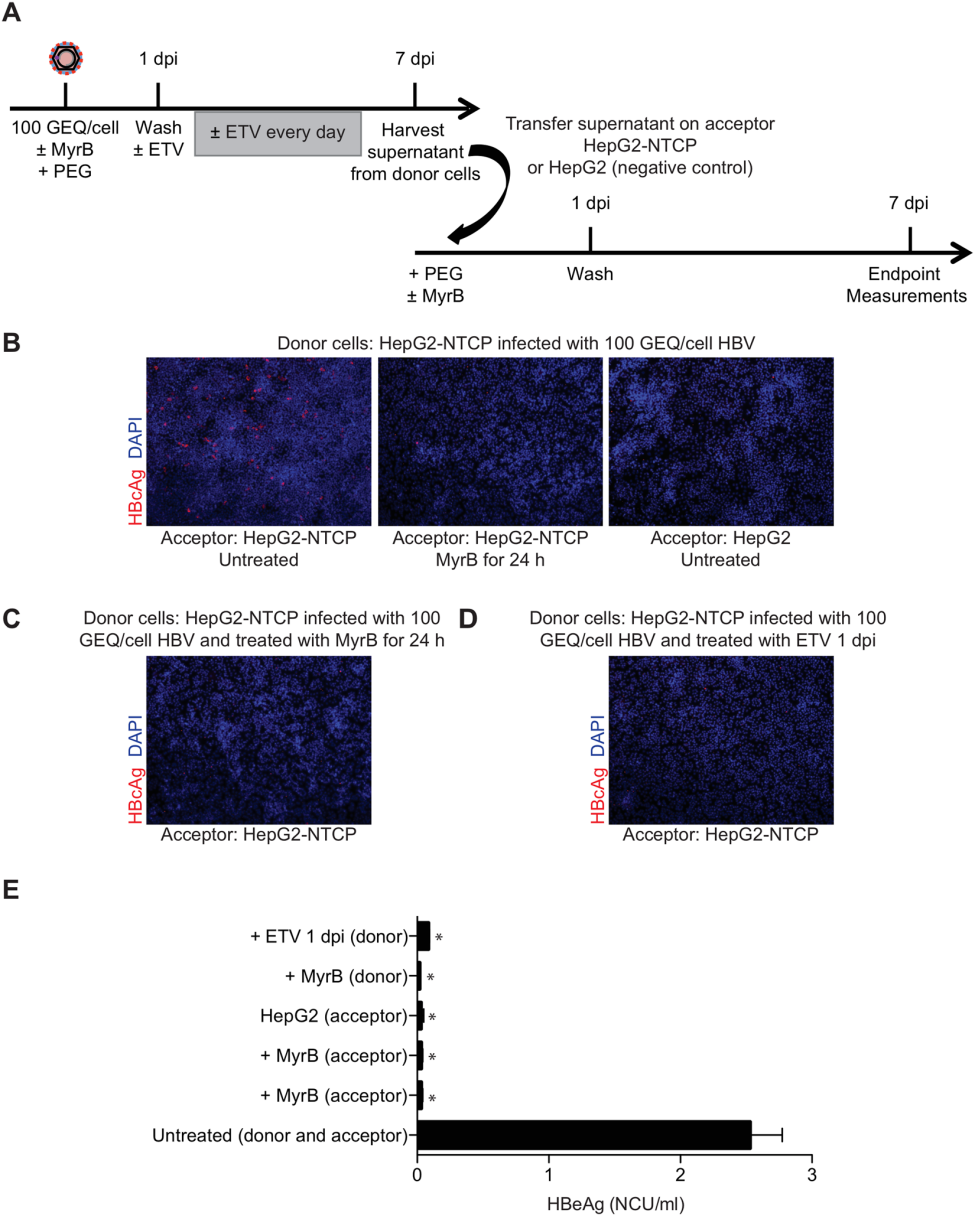
HepG2-NTCP cells produce infectious HBV. (**A**) Schematic of experimental design. (**B**) Immunofluorescence microscopy to detect HBcAg in infected HepG2-NTCP cells. HepG2-NTCP cells were infected with 100 GEQ/cell of HBV in the presence of 2% DMSO and 4% PEG. One day post-infection fresh medium containing 2% DMSO was added. At 7 dpi supernatant from donor cells was transferred to naïve HepG2-NTCP cells (acceptor). On the day of transfer, naïve cells were treated with 4% PEG, 2% DMSO in the presence of absence of 500 nM MyrB for 24 h. 2% DMSO was included through the entire course of infection. As controls (acceptor) HepG2 cells that do not express NTCP and HepG2-NTCP cells treated with MyrB were used. (**C**) HepG2-NTCP cells were infected with HBV in the presence of MyrB (donor) and supernatant was transferred on naïve HepG2-NTCP cells (acceptor). On the day of transfer cells were treated with PEG for 24 h. This experiment was used as a control for carryover inoculum. (**D**) HepG2-NTCP cells were infected with HBV following the same protocol with panel B (donor). One day post-infection ETV was added and kept until 7 dpi. The supernatant was then transferred on naïve HepG2-NTCP cells (acceptor). (**E**) Quantification of HBeAg levels released in supernatant from acceptor cells. Six biological replicates were performed and data are shown as means ± s.d. (one-way ANOVA *P < 0.001).

There is no evidence to date to that HBV spreads in HepG2-NTCP cell culture systems. To understand why, we tested whether HBV-infected HepG2-NTCP cells release infectious virus. The experimental design is shown in Fig. 3A. Using the protocol described above, we infected HepG2-NTCP cells in a 24-well plate with 100 GEQ/cell of HBV for one day in the presence of 2% DMSO and 4% PEG. One day post-infection we aspirated the inoculum and after 5 PBS washes we added fresh medium with 2% DMSO but without PEG. At 7 dpi we collected cell supernatants and transferred to naïve HepG2-NTCP cells in the presence of 2% DMSO. We also included MyrB in supernatant transfer experiments to rule out the possibility that infected cells were carried over from the primary infection (Fig. 3B). As an additional control we used HepG2 cells that do not express NTCP (Fig. 3B). To rule out the possibility that infection resulted from carryover of the initial inoculum, we used MyrB in the primary infection and then supernatant was transferred on naïve HepG2-NTCP cells (Fig. 3C). Moreover, we included treatment with the polymerase inhibitor entecavir (ETV) during the primary infection at one day post-infection and 7 dpi we transferred the supernatant on naïve HepG2-NTCP cells (Fig. 3D). The final concentration of ETV was 370 nM, which is 100 times the EC50 concentration as described before^17^.

As shown by HBcAg staining in Fig. 3B–D and HBeAg quantification in Fig. 3E, supernatants collected from HepG2-NTCP cells 7 dpi contained infectious HBV. From this it was clear that HBV-infected HepG2-NTCP cells produce infectious virus; however, it was still unclear why HBV fails to spread in culture.

### Maintaining PEG in culture medium enhances HBV infection in HepG2-NTCP cells

Given that HBV-infected HepG2-NTCP cells produce infectious virus and that PEG significantly increases infection, we reasoned that maintaining PEG in culture medium after the initial infection may promote virus spread. We therefore tested whether cells tolerate prolonged incubation with 4% PEG and whether it affects HBV infection.

**Figure 4:**
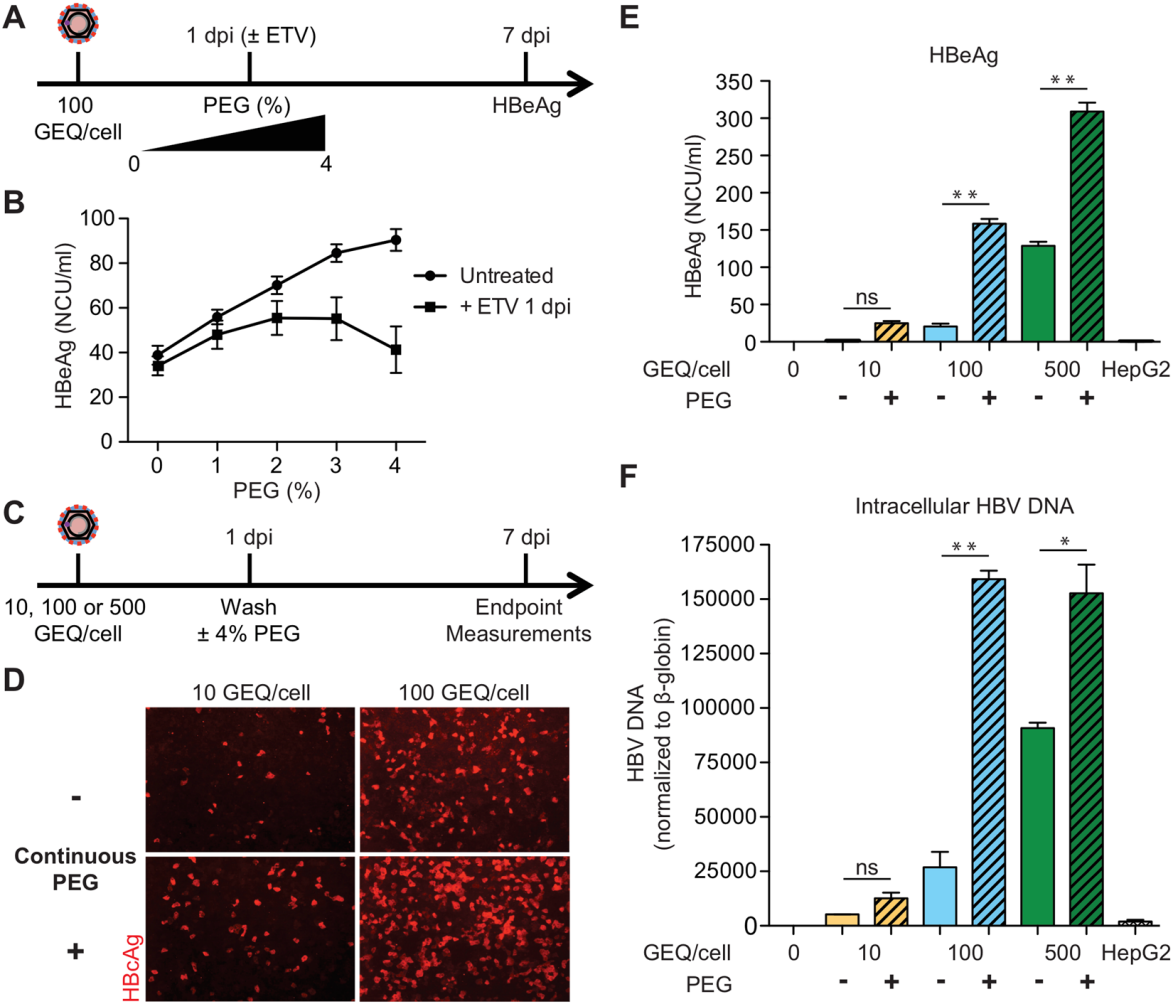
Continuous incubation with PEG results in enhanced efficiency of HBV infection in HepG2-NTCP cells. (**A**) Schematic of experimental design for panel B. (**B**) Cells were infected with 100 GEQ/cell in the presence of 4% PEG. Then, 1 dpi cells were washed and medium was added in the presence of different concentrations of PEG (0–4%) with or without 370 nM ETV until the end of the experiment. Secreted HBeAg was measured 7 dpi. (**C**) Schematic of experimental design for panels D–F. (**D**) Immunofluorescence microscopy of HBcAg in HepG2-NTCP cells infected with 10 or 100 GEQ/cell. PEG was added during the infection and 1 dpi was either removed or kept in the medium until the end of the experiment. (**E**) HBeAg levels and (**F**) intracellular HBV DNA in HepG2-NTCP cells infected with 10, 100 or 500 GEQ/cell with or without continuous treatment with PEG. Inoculum concentrations are shown in different colors and the presence of PEG is denoted by patterned backgrounds. HepG2 cells without NTCP infected with 500 GEQ/cell were used as a negative control. HBV DNA levels were normalized to human β-globin and were plotted as a fold change relative to uninfected cells. At least three biological replicates were performed and data are shown as means ± s.d. (one-way ANOVA, *P < 0.01; **P < 0.001; n.s., not significant).

In Fig. 4, we show that maintaining PEG in the culture medium after removing virus inoculum increased HBV infection in a dose dependent way (Fig. 4B). Specifically, the increase was by 2- to 20-fold as measured by HBcAg staining (Fig. 4D), HBeAg ELISA (Fig. 4E), and intracellular HBV DNA (Fig. 4F). The effect was most pronounced when cells were infected with low virus concentrations (compare 10 and 100 vs. 500 GEQ/cell). Since the initial viral inoculum was removed after day one, we speculated that the increased infection at 7 dpi resulted from viral spread. To support this, treatment with ETV one day post-infection reduced the effects of PEG as shown by decreased HBeAg levels in Fig. 4B. To better determine the timing of the effect of PEG on enhanced HBV infection efficiency we infected cells with low virus concentration (20 GEQ/cell) and compared the cumulative HBeAg levels with or without the addition of 4% PEG 1 dpi at different time points. As shown in Supplementary Figure S1, HBeAg levels are higher at all time points in the PEG treated cells.

### Enhanced HBV infection in HepG2-NTCP cells is NTCP dependent

**Figure 5:**
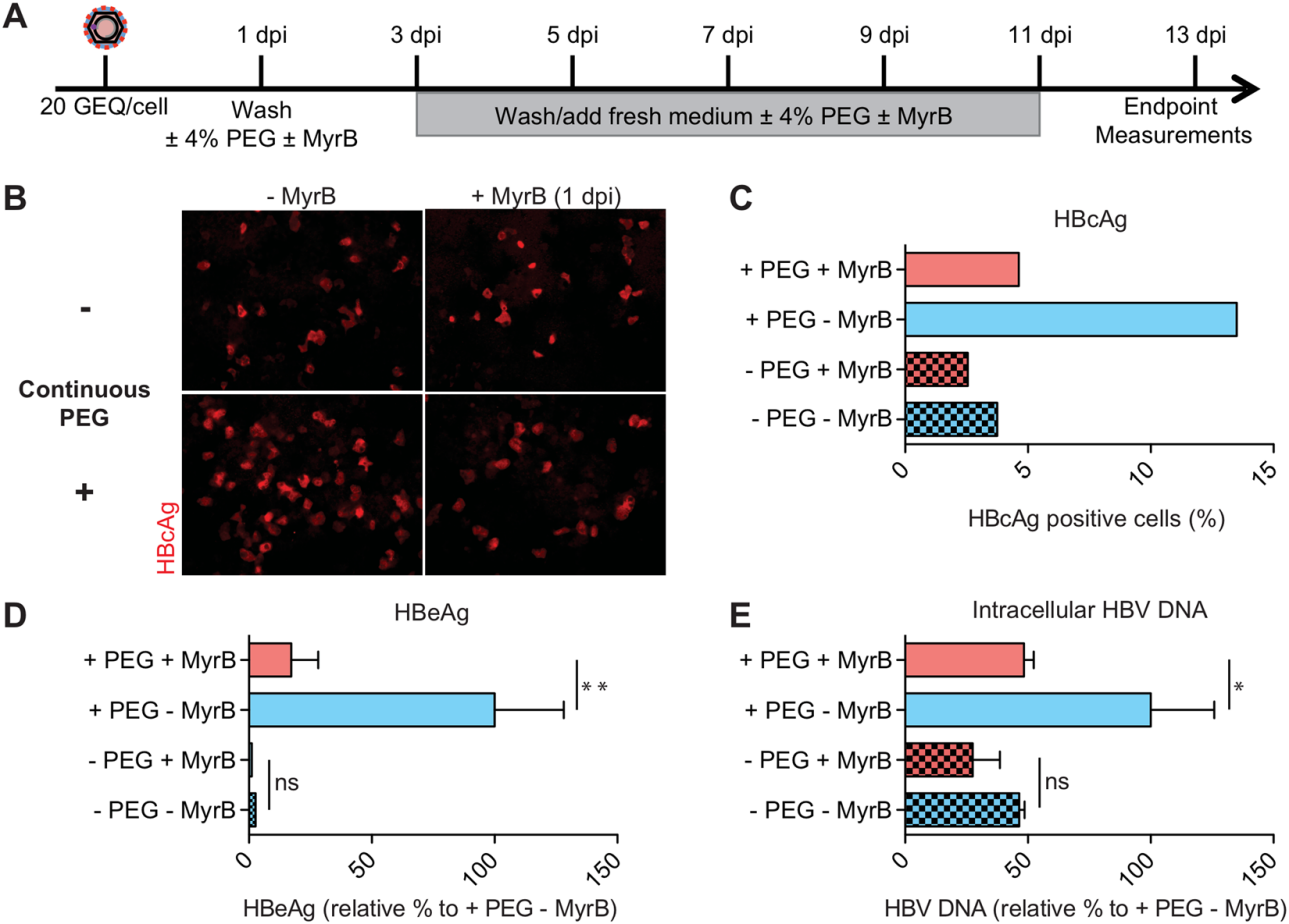
Enhanced HBV infection of HepG2-NTCP cells with continuous PEG treatment is NTCP-mediated (**A**) Schematic of experimental design. (**B**) Immunofluorescence microscopy of HBcAg in HepG2-NTCP cells infected with 20 GEQ/cell 13 dpi. PEG was added during the infection and was either removed or kept in the medium one day after infection with or without MyrB. Medium was changed every two days and PEG and MyrB were replenished in the respective wells. Continuous treatment with PEG promotes HBV spread (lower left panel). When MyrB was added the effect of spread was reduced (lower right panel). (**C**) Quantification of HBcAg positive cells 13 dpi. (**D**) HBeAg levels in supernatant, and (**E**) intracellular HBV DNA levels from at least three biological replicates are shown as means ± s.d. (one-way ANOVA, *P < 0.05; **P < 0.001; n.s., not significant). Data are represented as relative % of the infected/untreated cells.

Having shown that infection increases over time when cells are maintained in PEG, we next tested whether the increased infection was receptor dependent. For this, we used MyrB as a control to block NTCP. As above, we infected cells for one day in the presence of PEG and then removed virus inoculum. We then cultured cells for an additional 12 days—with medium changes every two days—in medium containing 4% PEG with and without MyrB. The experimental design is shown in Fig. 5A.

As shown in Fig. 5B–E, we found that treating cells with MyrB for 12 days decreased the number of HBcAg-positive cells (Fig. 5B,C), the amount of HBeAg secreted into culture medium (Fig. 5D), and the amount of intracellular HBV DNA (Fig. 5E). This suggests that the increased frequency of HBV infection in HepG2-NTCP cells upon prolonged PEG treatment is receptor-dependent.

### Co-culture system demonstrates that continuous PEG treatment facilitates HBV spread in HepG2-NTCP cells

**Figure 6:**
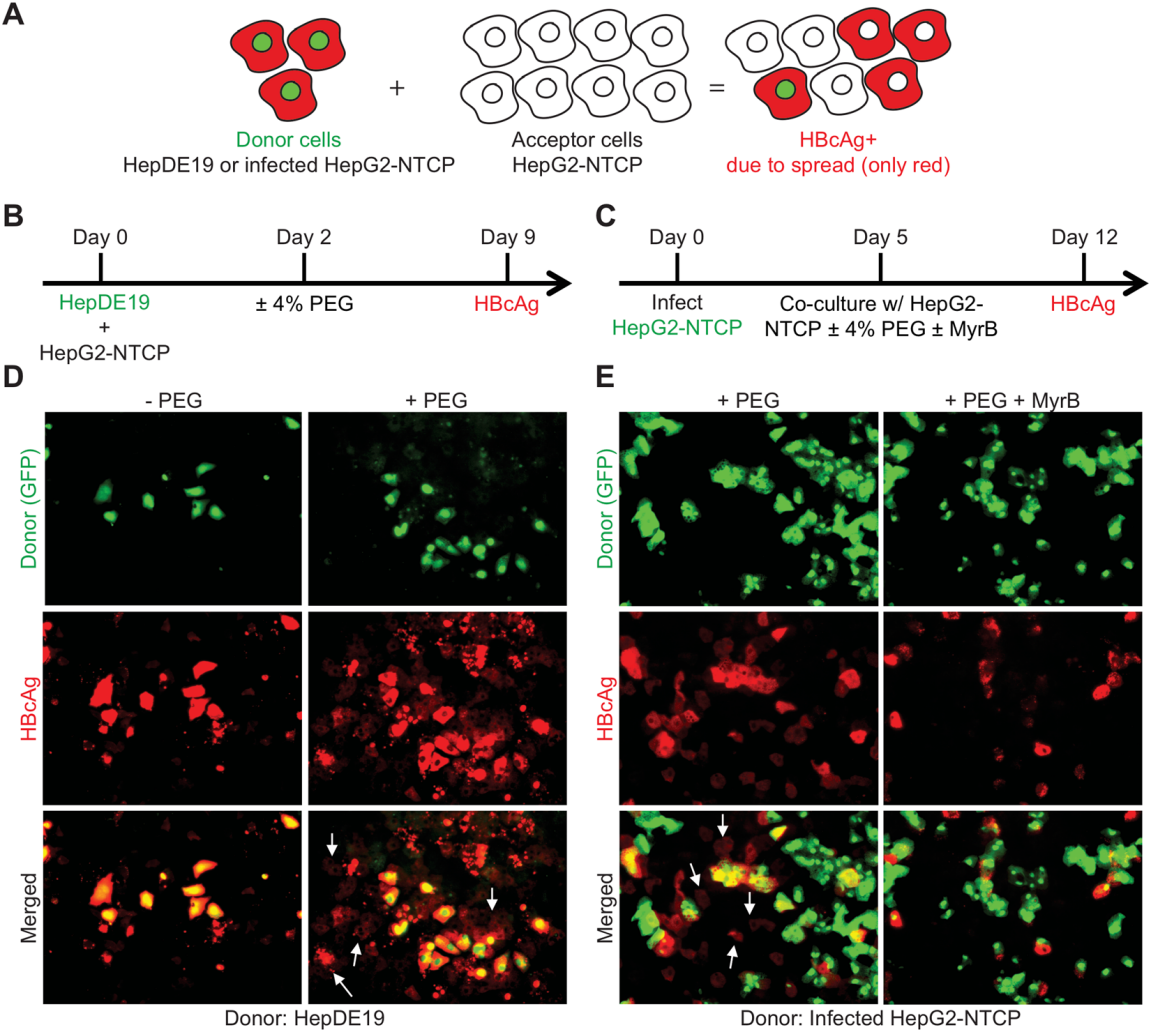
Co-culture systems with PEG support HBV spread in HepG2-NTCP cells. (**A**–**C**) Schematics of experimental designs of two co-culture systems of HBV donor and acceptor cells in the presence of PEG. (**D**) HBV donor cells (HepDE19-GFP) were co-cultured with acceptor HepG2-NTCP cells at a 1:10 ratio. The cells were treated with or without 4% PEG. After 7 days the cells were fixed and stained for HBcAg (red). Arrows indicate newly infected cells as a result of virus spread. (**E**) HBV donor cells (infected HepG2-NTCP-GFP) were co-cultured with acceptor HepG2-NTCP at 1:10 ratio in the presence of 4% PEG with or without MyrB. After 7 days the cells were fixed and stained for HBcAg (red). Quantification of HBcAg positive cells due to spread is shown in Table 2.

Next, to further confirm that continuous PEG treatment facilitates HBV spread, we co-cultured uninfected HepG2-NTCP cells (acceptor cells) with HBV-producing cells (donor cells) and tested whether acceptor cells became infected. For donor cells, we used either HepDE19 cells^16^ or HBV-infected HepG2-NTCP cells. In both cases the donor cells expressed GFP as a marker and we co-cultured cells at a ratio of 1:10—one donor cell for every 10 acceptor cells. Then, after seven days, we stained cells for HBcAg to monitor HBV spread. A schematic of the co-culture systems is shown in Fig. 6A and the experimental designs are shown in Fig. 6B,C. Cells were treated with 2% DMSO throughout the experiment in the presence or absence of 4% PEG.

Figure 6D and Table 2 show the results from co-culturing GFP-tagged HepDE19 cells with naïve HepG2-NTCP cells in the presence or absence of PEG. Overall, HBcAg fluorescence intensity is higher in HepDE19 cells, but approximately 57% of neighboring and non-neighboring HepG2-NTCP acceptor cells were HBcAg positive. Therefore, like previous experiments, the results show that PEG facilitates HBV spread.

**Table 2:** HBV spread in co-culture systems.

Infected HepG2-NTCP-GFP co-cultured with HepG2-NTCP (+PEG ± MyrB)		
	+ PEG	+ PEG; + MyrB
Infected cells due to spread (%)	7.2	0.6
Fold change	1	12
**HBV producing HepDE19-GFP co-cultured with HepG2-NTCP (±PEG)**		
	**−PEG**	**+PEG**
Infected cells due to spread (%)	11	57
Fold change	1	5

Figure 6E and Table 2 show the results from co-culturing GFP-tagged pre-infected HepG2-NTPC cells with naïve HepG2-NTCP cells in medium containing 2% DMSO, 4% PEG with and without 500 nM MyrB. The GFP-expressing HepG2-NTCP donor cells were not 100% infected when we co-cultured cells, which explains why some GFP-positive cells are HBcAg negative. Overall, we observed a 12-fold increase in the number of GFP-negative, HBcAg positive cells in the untreated vs. MyrB-treated co-cultures, further indicating that HBV spreads in culture.

### Continuous treatment with PEG enhances infection with HBV clinical isolates

**Figure 7:**
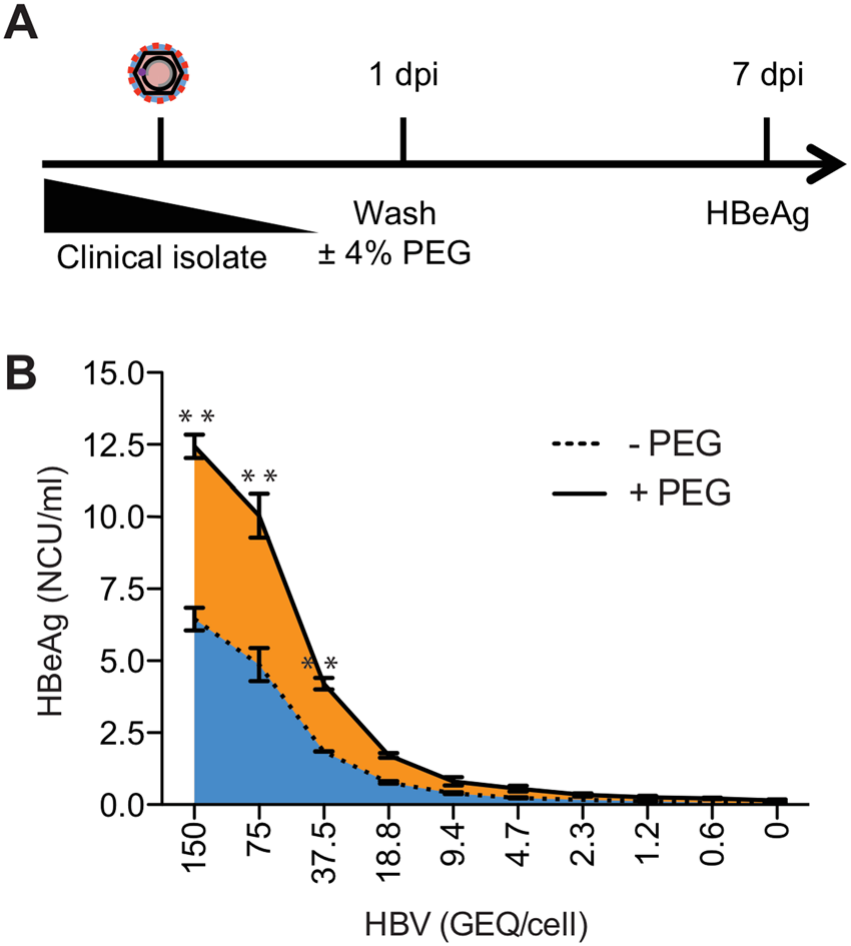
Continuous treatment with PEG enhances infection with HBV clinical isolates. (**A**) Schematic of experimental design. (**B**) HepG2-NTCP cells were infected with serial dilutions of a genotype D HBV patient serum (0.6–150 GEQ/cell) in the presence of 4% PEG. One day post-infection the cells were washed and cultured in either the absence (dashed line) or presence (solid line) of PEG for an additional 6 days before harvesting the supernatants for HBeAg ELISA. Data from three biological replicates are shown as means ± s.d. (one-way ANOVA, **P < 0.001).

To test our system in a more physiological context, we used serially diluted sera (0.6–150 GEQ/cell) from a genotype D HBV-infected patient to infect HepG2-NTCP cells in culture. As usual, cells were infected for one day, washed, then cultured in medium with and without PEG. As shown in Fig. 7B, the amount of HBeAg in culture supernatants 7 dpi correlated with the amount of virus inoculum used for infection. Consistent with virus spread, we observed 2 to 3-fold higher levels of HBeAg when cells were continuously treated with PEG. Thus, like tissue culture derived virus, culturing cells in the presence of PEG facilitates spread of HBV from patient serum.

### ISG20 and tetherin restrict HBV spread

We next sought to test the utility of this system for studying key questions in the HBV life cycle. HBV is thought to be a stealth virus that escapes the innate immune response^18^. There is, however, data from chimpanzee studies that suggest a pre-existing type I interferon (IFN) response due to HCV infection limits subsequent HBV infection^19^. We therefore chose to validate our HBV cell culture system by studying two type I IFN-stimulated genes (ISGs), ISG20 and BST2 (also known as tetherin), that have been shown to inhibit HBV *in vitro*. ISG20 inhibits HBV by several mechanisms including degradation of viral RNAs and by directly binding a viral RNA structure known as epsilon required for RNA encapsidation^20,21^. Tetherin has been shown to inhibit HBV secretion; making it an important test of our HBV spread system^22,23^.

**Figure 8:**
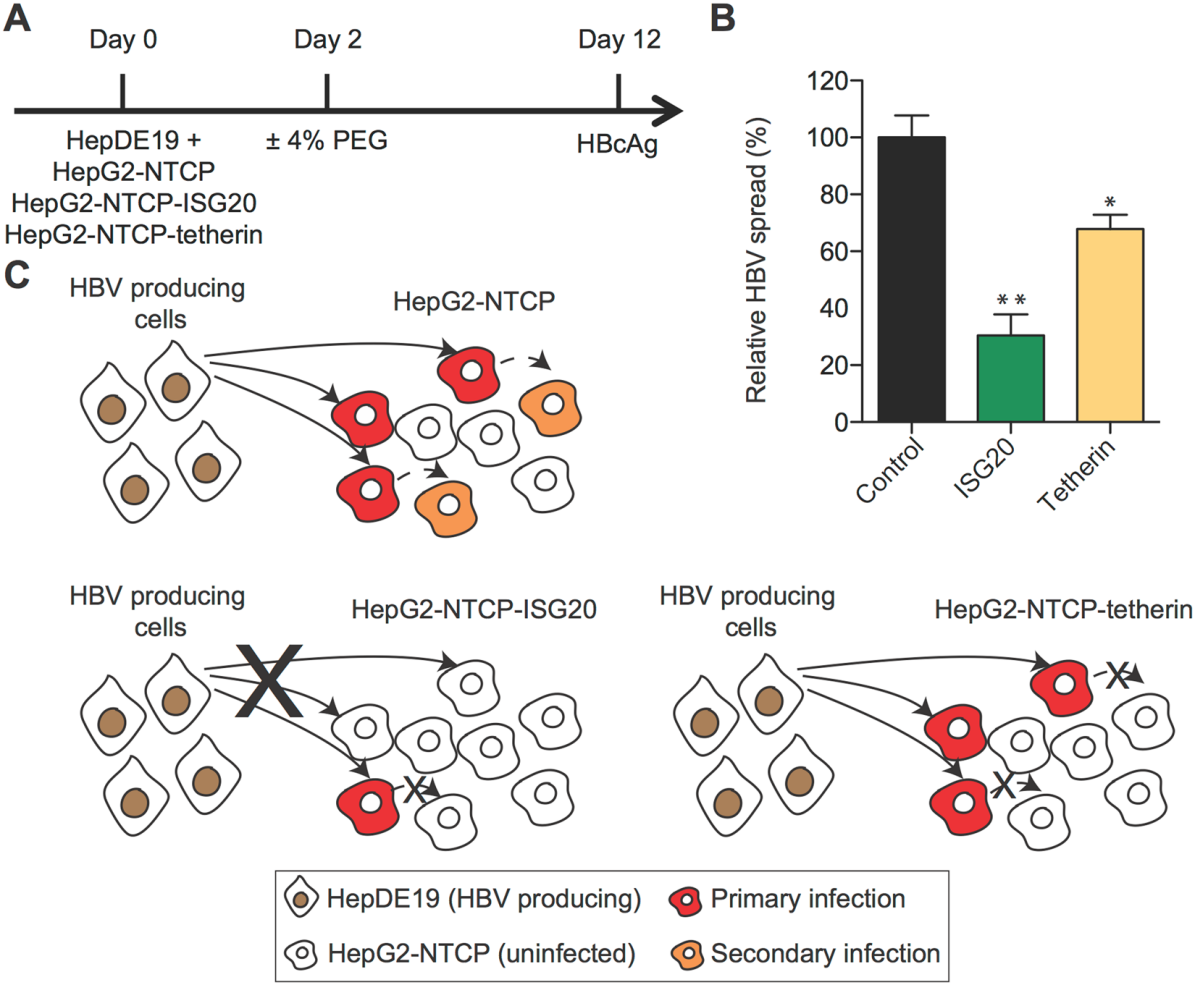
ISG20 and tetherin restrict HBV spread in HepG2-NTCP cells. (**A**) Schematic of experimental design. (**B**) Co-culture of HBV donor cells (HepDE19) with acceptor cells (HepG2-NTCP stably overexpressing ISG20 or tetherin or empty/control) at a 1:10 ratio. PEG was added in the co-culture for an additional 10 days. High content imaging analysis of HBcAg positive cells was performed using ImageXpress. Data from five biological replicates are shown as means ± s.d. (one-way ANOVA, *P < 0.05; **P < 0.001). As 100% spread we set the spread in HepG2-NTCP-empty cells. **(C)** Schematic representation of restriction of HBV spread in ISG20 or tetherin expressing cells.

To study these ISGs in the context of virus spread, we generated three HepG2-NTCP cell lines expressing either ISG20, tetherin, or an empty vector control. We then co-cultured these cells with HBV-producing HepDE19 cells in the presence of PEG for 10 days to allow virus spread and quantified the number of HBcAg positive cells in the HepG2-NTCP populations. A schematic of the experiment is shown in Fig. 8A. Figure 8B shows the level to which HBV spreads to the HepG2-NTCP cells. For comparison, we set values from the empty vector control population to 100% and found that tetherin inhibited HBV infection by approximately 30% and ISG20 inhibited HBV infection by approximately 70%.

This strongly suggests that ISG20 restricts HBV spread to HepG2-NTCP cells (Fig. 8C). Interestingly, viral spread to tetherin-expressing HepG2-NTCP cells in the presence of PEG was only marginally affected compared to the control cells (about 30% reduction) (Fig. 8B). Given the role of tetherin in restriction of HBV budding and release from infected cells^22^, we speculate that while ISG20 likely inhibits both primary infection and spread, tetherin only inhibits spread (Fig. 8C). However, the presence of tetherin restricted further secondary infection/spread, resulting in an overall modestly reduced infection rate that was less pronounced as compared to ISG20-expressing cells (Fig. 8C).

In conclusion, we demonstrate that a simple yet significant modification to an already in-use cell culture system enables HBV spread and allows, for the first time, the complete HBV life cycle to be studied in cell culture. Until now, viral spread has not been detected in NTCP-overexpressing HepG2 cells^8,9,11,15^, but we show that maintaining PEG in cell culture medium increases infection by at least one order of magnitude, largely due to virus spread. This simple adaptation to existing HBV infection protocols will allow open questions regarding HBV biology from virus entry to spread to be addressed in already existing cell culture systems. Moreover, these methods provide a new platform to identify and evaluate anti-HBV agents that aim to cure chronic HBV infection.

## Methods

### Generation of HepG2-NTCP cells

Human NTCP cDNA was cloned into the neomycin resistance co-expressing lentiviral vector, pWPI-neo. Low passage HepG2 cells were transduced with NTCP-expressing lentiviruses and selected with 500 μg/ml G418. Once NTCP expression was confirmed by immunofluorescence staining, several single cell clones were subcultured and tested for their permissiveness to HBV. One of those clones (clone 7) was shown to support the highest HBV infection efficiency as shown by HBcAg staining and was used throughout this study. To generate HepG2-NTCP-ISG20 and HepG2-NTCP-tetherin cells we transduced HepG2-NTCP cells with lentiviruses expressing the ISG of interest (pSCRPSY lentiviral expression vector)^24,25^ and selected the transduced cells with puromycin.

### Preparation of HBV stock

HepDE19 cells^16^ (generously provided by Dr. Haitao Guo, Indiana University) were cultured on collagen-coated T175 flasks in Dulbecco’s modified minimal essential medium (DMEM) supplemented with 3% FBS and 0.1 mM non-essential amino acids (NEAA). The cells were cultured in the absence of tetracycline to induce HBV replication. After seven days in culture without tetracycline to induce HBV expression, supernatant was collected every other day for two weeks and fresh medium was added. After each collection, medium was spun down at 1,000 × g at 4 °C to remove any cell debris and passed through a 0.22 μm filter. During this process, the medium was kept at 4 °C. At the end of all the collections the medium was concentrated 100-fold via centrifugation using Centricon Plus-70 centrifugal filter devices (Millipore-Sigma, Billerica, MA). The concentrated virus stock was aliquoted and stored at −80 °C. One of the aliquots was used to determine the HBV genomes/ml with a TaqMan-based qPCR method.

### HBV infection

HepG2-NTCP cells were seeded in either 96-well or 24-well collagen-coated plates in DMEM supplemented with 10% FBS and 0.1 mM NEAA. The cells were confluent the next day and the medium was changed to medium consisting of 3% FBS, 0.1 mM NEAA, and 2% DMSO. After 24 h of DMSO treatment the cells were infected with the concentrated HBV stock at the indicated GEQ/cell. The stock was diluted in inoculum media which is DMEM supplemented with 3% FBS, 0.1 mM NEAA, 2% DMSO, and 4% PEG (Sigma Aldrich, St. Louis, MO). The inoculum media was made fresh by initially dissolving PEG in DMEM supplemented with 3% FBS and 0.1 mM NEAA for a final PEG concentration of 8%. This solution was filtered sterilized and mixed with DMEM supplemented with 3% FBS and 0.1 mM NEAA, DMSO for final concentration of 2%, and virus stock at a volume respective to the desired GEQ/cell infection. The final concentration of PEG was 4% unless otherwise stated. The inoculum volume was adjusted to the plate format (50 μl for 96-well plates and 500 μl for 24-well plates). To minimize cell proliferation, we used low serum concentration (3% FBS) and 2% DMSO until the end of the experiment. The cells were spinoculated for 1 h by centrifugation at 1,000 × g at 37 °C^10^. After 24 h, inoculum was removed using vacuum aspiration, cells were washed five times with PBS and 100 μl (96-well plate) or 500 μl (24-well plate) of fresh medium DMEM supplemented with 3% FBS and 0.1 mM NEAA was added in the presence of 2% DMSO. Depending on the experiment, 4% or no PEG was added to the medium and the cells were cultured for the indicated time before harvesting and further analysis. Plasma from unidentified HBV-infected individuals was obtained from the Red Cross and was used to infect cells in a similar fashion as the tissue culture derived HBV stock. However, 1 ml plasma was pre-treated with 25 mM CaCl_2_ for 2 h at 37 °C and thoroughly spun down at 10,000 × g to remove coagulation factors. The supernatant was transferred to a new tube and was incubated for an additional 30 min at 37 °C and spun down at 10,000 × g to remove any remaining debris. The clarified sample was mixed with dilution media depending on the desired GEQ/cell for a final concentration of 4% PEG and 2% DMSO. Part of the clarified sample was used for DNA extraction and determination of the GEQ/ml using a Taqman-based method described later in the methods section. To define the genotype, the extracted DNA was used for PCR amplification with the following primers: (F) 5′CTCCACCAATCGGCAGTC3′ and (R) 5′AGTCCAAGAGTCCTCTTATGTAAGACCTT3′. The PCR product was sequenced using the following primer: 5′CCTCTGCCGATCCATACTGCGGAAC3′ and the genotype was determined using NCBI genotyping online tool at: http://www.ncbi.nlm.nih.gov/projects/genotyping/formpage.cgi.

### Immunofluorescence

Cells were fixed in 4% paraformaldehyde for 20 min at room temperature, washed with PBS and permeabilized with 0.1% Triton X-100 for 10 min. After washing with PBS and blocking in 5% goat serum (Jackson ImmunoResearch, West Grove, PA) for at least 1 h, cells were incubated overnight at 4 °C in a 1:500 dilution of primary antibodies in PBS with 5% goat serum [rabbit anti-human NTCP (Sigma Aldrich); rabbit anti-HBV core (Austral Biologicals, San Ramon, CA)]. Secondary antibodies were goat anti-rabbit AlexaFluor 488 or 594 (Life Technologies, Carlsbad, CA) at a dilution of 1:1000. Nuclei were stained with DAPI. Cells were imaged using a Nikon Eclipse TE300 fluorescent microscope and processed with ImageJ. For high-content imaging analysis ImageXpress Micro XLS (Molecular Devices, Sunnyvale, CA) was used.

### Intracellular HBV DNA

Infected cells were pelleted, washed with PBS and stored at −80 °C. DNA was extracted using a QIAamp DNA blood mini kit (Qiagen, Hilden, Germany) according to the manufacturer’s protocol. Total HBV DNA was determined with qPCR analysis as previously described^26^. Briefly, the qPCR was performed using a TaqMan Universal PCR Master Mix (Applied Biosystems, Foster City, CA) and the following primers and probe: 5′-CCGTCTGTGCCTTCTCATCTG-3′ (sense), 5′-AGTCCAAGAGTCCTCTTATGTAAGACCTT-3′ (anti sense), 5-/56 FAM/CCGTGTGCA/ZEN/CTTCGCTTC ACCTCT GC/3IABkFQ/-3 (probe). PCR was done using a Roche LightCycler 480 PCR machine and the HBV copies/sample were calculated based on a standard curve composed from 2xHBV plasmid in a concentration range of 10^9^–101 copies.

### HBeAg chemiluminescence immunoassay

For quantitative analysis of secreted HBeAg, 50 μl of supernatants were loaded into 96-well plates of a chemiluminescence immunoassay (CLIA) kit according to the manufacturer’s instructions (Autobio Diagnostics Co., Zhengzhou, China). Plates were read using a FLUOstar Omega luminometer. Concentrations are expressed in national clinical units per milliliter (NCU/ml).

### Data availability statement

No datasets were generated or analyzed during the current study.

## Electronic supplementary material

Supplementary Information
