# Supplementary material for: A robust cell culture system supporting the complete life cycle of hepatitis B virus
Source: Sci Rep. 2017 Nov 30;7:16616. doi: 10.1038/s41598-017-16882-5 (PMC5709435; doi:10.1038/s41598-017-16882-5)
Supplement: Supplementary file 1 — Supplementary Information [file 41598_2017_16882_MOESM1_ESM.pdf]

Supplementary Material

A robust cell culture system supporting the complete life cycle of hepatitis B virus

Eleftherios Michailidis[1], Jonathan Pabon[1, 8], Kuanhui Xiang[1, 2, 8], Paul Park[1], Vyas Ramanan[3], Hans-Heinrich Hoffmann[1], William M. Schneider[1], Sangeeta N. Bhatia[4, 5], Ype P. de Jong[1, 6], Amir Shlomai[7], Charles M. Rice[1, *]

[1]Laboratory of Virology and Infectious Disease, The Rockefeller University, New York, NY, USA.

[2]Department of Microbiology and Center of Infectious Disease, School of Basic Medical Sciences, Peking University Health Science Center, Beijing, China.

[3]Department of Health Sciences and Technology, Massachusetts Institute of Technology, Cambridge, MA.

[4]Institute for Medical Engineering and Science, David H. Koch Institute for Integrative Cancer Research, Department of Electrical Engineering and Computer Science, Massachusetts Institute of Technology, Cambridge, MA.

[5]Howard Hughes Medical Institute, Chevy Chase, MD.

[6]Division of Gastroenterology and Hepatology, Weill Cornell Medical College, New York, NY, USA.

[7]Department of Medicine D and the Liver Institute, Rabin Medical Center, Belinson Hospital, Petach-Tikva and Sackler Faculty of Medicine, Tel Aviv University, Tel Aviv, Israel.

[8]Equal contribution.

* For correspondence: ricec@rockefeller.edu

Supplementary Figure S1

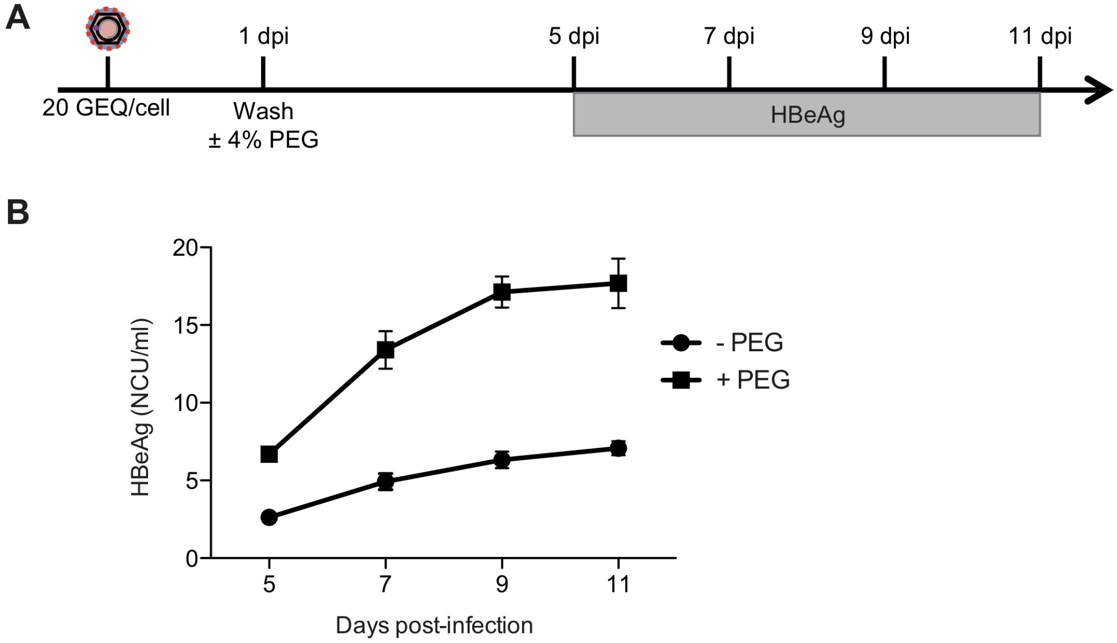

Figure S1. Time course of enhanced HBV infection efficiency in the presence of continuous PEG treatment. (A) Schematic of experimental design for panel B. **(B)** Cells were infected with 20 GEQ/cell in the presence of 4% PEG. Then, 1 dpi cells were washed and medium was added in the presence or absence of 4% PEG. After the indicated time points (5, 7, 9 or 11 dpi) supernatants were harvested and secreted HBeAg was measured. Four biological replicates were performed and data are shown as means ± s.d.
